# Self-sampling tools to increase cancer screening among underserved patients: a pilot randomized controlled trial

**DOI:** 10.1093/jncics/pkad103

**Published:** 2023-12-07

**Authors:** Jennifer L Moss, Juliette Entenman, Kelsey Stoltzfus, Jiangang Liao, Tracy Onega, Paul L Reiter, Lisa M Klesges, George Garrow, Mack T Ruffin

**Affiliations:** Department of Family and Community Medicine, Penn State College of Medicine, The Pennsylvania State University, Hershey, PA, USA; Department of Public Health Sciences, Penn State College of Medicine, The Pennsylvania State University, Hershey, PA, USA; Department of Family and Community Medicine, Penn State College of Medicine, The Pennsylvania State University, Hershey, PA, USA; Department of Family and Community Medicine, Penn State College of Medicine, The Pennsylvania State University, Hershey, PA, USA; Department of Public Health Sciences, Penn State College of Medicine, The Pennsylvania State University, Hershey, PA, USA; Huntsman Cancer Institute, Salt Lake City, UT, USA; Department of Population Health Sciences, School of Medicine, University of Utah, Salt Lake City, UT, USA; Department of Health Behavior and Health Promotion, College of Public Health, The Ohio State University, Columbus, OH, USA; Department of Surgery, Washington University School of Medicine in St Louis, St Louis, MO, USA; Primary Health Network, Sharon, PA, USA; Department of Family and Community Medicine, Penn State College of Medicine, The Pennsylvania State University, Hershey, PA, USA

## Abstract

**Background:**

Screening can reduce cancer mortality, but uptake is suboptimal and characterized by disparities. Home-based self-sampling can facilitate screening for colorectal cancer (with stool tests, eg, fecal immunochemical tests) and for cervical cancer (with self-collected human papillomavirus tests), especially among patients who face barriers to accessing health care. Additional data are needed on feasibility and potential effects of self-sampling tools for cancer screening among underserved patients.

**Methods:**

We conducted a pilot randomized controlled trial with patients (female, ages 50-65 years, out of date with colorectal and cervical cancer screening) recruited from federally qualified health centers in rural and racially segregated counties in Pennsylvania. Participants in the standard-of-care arm (n* *=* *24) received screening reminder letters. Participants in the self-sampling arm (n* *=* *24) received self-sampling tools for fecal immunochemical tests and human papillomavirus testing. We assessed uptake of screening (10-week follow-up), self-sampling screening outcomes, and psychosocial variables. Analyses used Fisher exact tests to assess the effect of study arm on outcomes.

**Results:**

Cancer screening was higher in the self-sampling arm than the standard-of-care arm (colorectal: 75% vs 13%, respectively, odds ratio = 31.32, 95% confidence interval = 5.20 to 289.33; cervical: 79% vs 8%, odds ratio = 72.03, 95% confidence interval = 9.15 to 1141.41). Among participants who returned the self-sampling tools, the prevalence of abnormal findings was 24% for colorectal and 18% for cervical cancer screening. Cancer screening knowledge was positively associated with uptake (*P* < .05).

**Conclusions:**

Self-sampling tools can increase colorectal and cervical cancer screening among unscreened, underserved patients. Increasing the use of self-sampling tools can improve primary care and cancer detection among underserved patients.

**Clinical Trials Registration Number:**

STUDY00015480.

In 2023, an estimated 24 080 women will die from colorectal cancer, and 4310 women will die from cervical cancer (1). The US Preventive Services Task Force recommends screening for colorectal cancer (2) and for cervical cancer (3) to reduce cancer mortality. However, screening participation falls short of national goals (4), particularly for patients with low health-care access (5-8) and health literacy (9). Notably, screening is lower in rural than urban counties (10,11), and these disparities are even wider in counties that are also racially segregated (12,13) [ie, with high spatial concentrations of a given racial group (14)].

One approach to increasing cancer screening is via tools that allow patients to collect a sample at home (ie, self-sampling), a strategy endorsed by the President’s Cancer Panel (15). For colorectal cancer screening, patients can use self-sampled stool tests (eg, fecal immunochemical tests) (2). For cervical cancer screening, there is not yet a self-sampling option, but self-sampled human papillomavirus (HPV) testing is routine in other countries (16-18), and it is accurate (19-21) and acceptable (22-24). These tests are effective for detecting precancers: sensitivity is approximately equal to 0.74 for fecal immunochemical tests compared with colonoscopy (25) and more than 0.96 for self-sampled HPV testing compared with colposcopy or biopsy (19). Self-sampling tools may be especially beneficial for increasing uptake among patients with low health-care access because they eliminate the need for an in-person clinical appointment, as well as for addressing other barriers to screening (eg, embarrassment) (11).

In this study, we conducted a pilot randomized controlled trial to assess the feasibility and potential effects of providing self-sampling tools to underserved patients. Participants were patients (female, ages 50-65 years) who were eligible but out of date with both colorectal and cervical cancer screening, recruited from federally qualified health centers in rural, racially segregated counties in Pennsylvania. These findings have implications for future policies and interventions to increase self-sampling for cancer screening.

## Methods

### Setting and participants

The setting was federally qualified health centers in rural, racially segregated counties in Pennsylvania. Rural counties were nonmetropolitan according to US Department of Agriculture’s rural urban continuum codes (26). Racially segregated counties were those with a dissimilarity index (27,28) for White vs non-White residents greater than the national median, according to 2014-2018 American Community Survey (29). Fourteen counties met these criteria, and our partner federally qualified health center has 9 clinics located in 6 of these counties. Together, these 9 clinics serve approximately 14 370 active patients.

Federally qualified health center patients were eligible for study participation if they lived in a rural and racially segregated county and had visited the federally qualified health center at least once in the previous 2 years. In addition, patients had to be eligible but out of date with screening for both colorectal (30) and cervical cancer (3) according to US Preventive Services Task Force guidelines in effect at the start of the trial. Specifically, patients had to be female sex and age 50-65 years [colorectal cancer screening guidelines have since expanded to start at age 45 years (2)]. Patients whose electronic health record indicated they had received screening within the recommended time interval were considered up to date and thus ineligible for the study; all others were considered out of date.

Exclusion criteria were having a partial or complete hysterectomy, a family history of colorectal cancer (ie, member of immediate family with colorectal cancer diagnosis age 40 years and younger), a personal history of cervical or colorectal cancer, and the inability to provide informed consent.

### Recruitment

**Figure 1.**
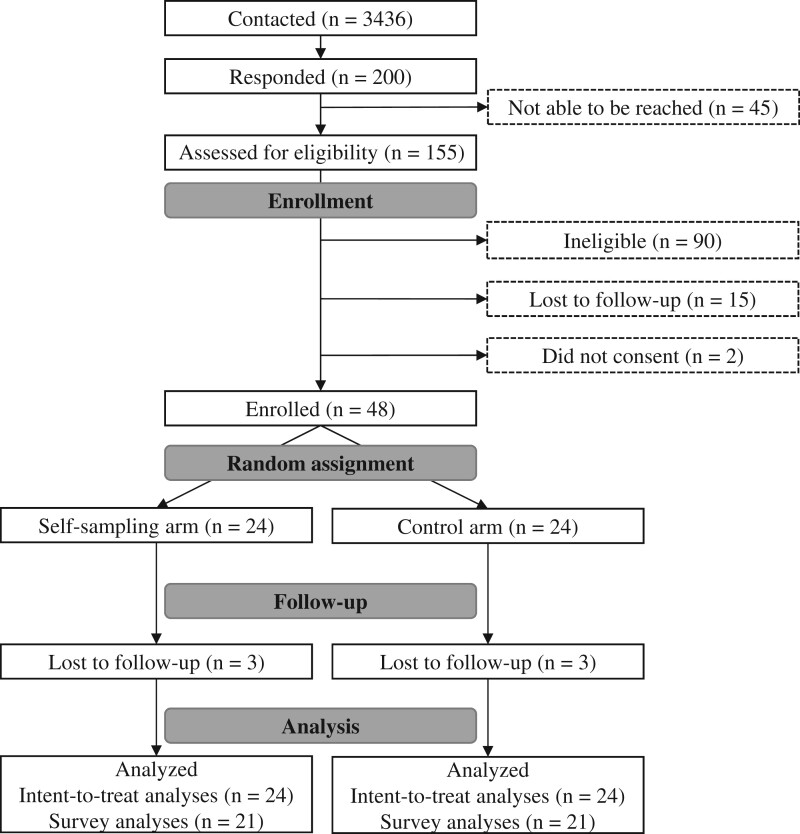
Participant flow chart for study procedures.

Federally qualified health centers personnel generated a list of potentially eligible patients using the criteria described above and then mailed patients an invitation letter describing the study. If patients did not respond, federally qualified health center personnel also sent e-mail and/or phone call messages to patients who had valid contact information. Patients could indicate interest in participating by returning a prestamped postcard or contacting the study office or federally qualified health center. Study staff contacted interested patients by phone to screen for eligibility, obtain verbal consent, and enroll participants. Of 3436 patients initially contacted, 200 responded to study invitations. We screened 155 patients of whom 90 were ineligible (primary reasons: 31 were younger than age 50 years, and 42 had a complete hysterectomy), and 48 were enrolled (see Figure 1). Recruitment took place February 2021 to December 2021, and all participants completed the study by March 2022.

Because of the exploratory nature of this study, we did not conduct an a priori power analysis. Our goal was to recruit 100 participants per arm, but because of slow accrual, we terminated recruitment after enrolling 24 participants per arm.

### Study procedures

After enrollment, study staff administered the baseline survey (115 items) over the phone. On average, participants took 26.8 minutes to complete the survey.

After completing the baseline survey, participants were randomly assigned to study arm (n* *=* *24 participants per arm) using an automated algorithm developed before data collection began. We chose individual-level rather than clinic-level random assignment because all study procedures occurred outside of the clinic, minimizing the potential for contamination across study arms (31). Next, study staff mailed participants a copy of the informed consent form and a $20 gift card to compensate them for their time completing the baseline survey, as well as the intervention materials for their study arm (see below).

Study staff administered the follow-up survey (72 items) over the phone 10 weeks after participant enrollment and then mailed participants a thank you letter and a $30 gift card. Of 48 participants, 6 (13%; n* *=* *3 from each arm) did not complete the follow-up survey. These participants did not differ from other participants in terms of baseline demographic or psychosocial variables (all *P* > .05).

#### Standard-of-care arm

These participants received a letter encouraging them to schedule an appointment for colorectal cancer screening and cervical cancer screening.

#### Self-sampling arm

These participants received a package containing 1-page, 2-sided educational fliers about 1) colorectal cancer and screening and 2) cervical cancer and screening; self-sampling tools and low-literacy instructions for 3) colorectal cancer screening (InSure ONE FIT) and 4) cervical cancer screening (Evalyn brush for HPV testing); and 5) a prestamped, padded envelope to mail the completed tools to Penn State Health Clinical Laboratories.

Participants who did not return their tools within 6 weeks (n* *=* *2) received a letter reminding them to do so. On average, participants returned their self-sampling tools 27.5 days (range = 14-76 days) after the study staff mailed the package.

All samples collected with the self-sampling tools were adequate for analysis. Returned fecal immunochemical tests were sent to a commercial lab for analysis. Returned self-sampled HPV tools were analyzed in house using procedures identical to those used for clinician-sampled HPV tests collected at Penn State Health, specifically, genotype analysis on the Roche cobas platform to identify HPV types 16, 18, and other high risk (types 31, 33, 35, 39, 45, 51, 52, 56, 58, 59, 66, or 68). Results of these analyses were communicated to study staff and the federally qualified health center, whose staff added the test results to each patient’s medical records. There was no cost incurred to participants for cancer screening.

For any participant whose screening test(s) indicated abnormal results, a physician called them within 7 days to discuss follow-up procedures. Patients who screened positive on fecal immunochemical test were referred to colonoscopy, and patients who screened positive for HPV were scheduled for gynecologic exam and a Pap test. Other patients did not receive clinical follow-up.

### Measures

Outcome measures were assessed during the 10-week follow-up survey. The co-primary outcomes were screening for 1) colorectal cancer and 2) cervical cancer via any modality. For participants who did not complete screening, we measured reasons for nonscreening as well as intentions to screen in the next year. Among participants in the self-sampling arm, we also assessed 1) return (yes vs no) and 2) results (abnormal vs normal) of each self-sampling tool.

Demographic and psychosocial variables that could impact screening were assessed during the baseline survey. Demographic characteristics included age (in years), race and ethnicity (non-Hispanic White vs other, including non-Hispanic African American or Black and multiracial participants), education (high school degree or less vs above high school degree), annual household income (<$50 000 vs ≥$50 000), insurance status (private, public vs other), and last-year preventive health-care check-up (no vs yes).

Psychosocial variables included health-care trust, cancer fatalism, and cancer screening knowledge. For health-care trust, we used the Trust in the Medical Profession instrument (11 items, range = 1-5, with higher scores indicating greater trust) (32). For cancer fatalism, we used 6 items from the National Cancer Institute’s Health Information National Trends Survey (range = 1-4, with higher scores indicating greater fatalism) (33). For cancer screening knowledge, we used 4 items from the Health Information National Trends Survey (range = 0-4, with higher scores indicating greater knowledge).

### Statistical analysis

First, we generated descriptive statistics for study variables (frequencies and proportions for categorical variables and means [SD] for continuous variables). To assess the success of random assignment, we tested for differences in baseline characteristics between arms using Fisher exact tests or Wilcoxon rank sum tests for categorical and continuous outcomes, respectively.

Next, we analyzed intervention effects by testing between-groups differences in screening for each cancer type using Fisher exact tests. We generated odds ratios (ORs) and 95% confidence intervals (CIs) with the Fisher exact tests to indicate the magnitude of between-groups differences. Because our randomization check did not detect any baseline differences between arms, we did not undertake adjusted analyses. Following intent-to-treat principles (34), we assumed that participants who did not complete the follow-up survey (n* *=* *6) were unscreened at follow-up.

Then, we analyzed self-sampling outcomes (return and results of the tests) for participants in the self-sampling arm only. We used Fisher exact tests to assess whether demographic and psychosocial variables were associated with self-sampling outcomes. Finally, we used Fisher exact tests to analyze whether psychosocial variables were associated with cancer screening (any modality).

Statistical analyses used a 2-sided *P* value of .05. Analyses were conducted using R. The Pennsylvania State University Human Research Protections Program approved data collection and analysis for this project. The trial protocol is registered at https://clinicaltrials.gov/study/NCT04471194 (35).

## Results

### Participant characteristics

On average, participants were aged 55.8 (SD = 4.3) years. Participants were predominantly (83%) non-Hispanic White, and most had annual household income less than $50 000 (67%) and public health insurance (56%) (Table 1).

**Table 1.** Baseline characteristics of participants (n=* *48) by study arm

Demographic variables	Standard-of-care arm No. (%)	Self-sampling arm No. (%)	*P*
Race and ethnicity			1.00
Non-Hispanic White	20 (83)	20 (83)	
Other^a^	4 (17)	4 (17)	
Education			1.00
High school degree or less	8 (33)	8 (33)	
Above high school degree	16 (67)	16 (67)	
Annual household income			.76
<$50 000	15 (62)	17 (71)	
≥$50 000	9 (38)	7 (29)	
Insurance status			.35
Private	10 (42)	6 (25)	
Public	11 (46)	16 (67)	
Other	3 (12)	2 (8)	
Last-year check-up			.14
No	2 (8)	7 (29)	
Yes	22 (92)	17 (71)	
Psychosocial variables			
Healthcare trust, mean (SD), range = 1-5	3.9 (0.9)	3.5 (0.9)	.13
Cancer fatalism, mean (SD), range = 1-4	2.0 (0.5)	2.3 (0.5)	.09
Cancer screening knowledge, mean (SD), range = 0-4	2.4 (0.8)	2.3 (0.9)	.96

a “Other” race and ethnicity includes n* *=* *2 non-Hispanic African American or Black and n* *=* *6 multiracial. Comparisons of demographic variables across study arms used Fisher exact tests, and comparisons of psychosocial variables used Wilcoxon rank sum tests.

### Cancer screening uptake

#### Uptake of both cancer screenings

Overall, 40% (19 of 48) of participants received colorectal and cervical cancer screenings during the follow-up period. Screening for both cancers was higher among participants in the self-sampling arm (75%; 18 of 24) than in the standard-of-care arm (4%; 1 of 24) (*P* < .001).

#### Uptake of colorectal cancer screening

**Figure 2.**
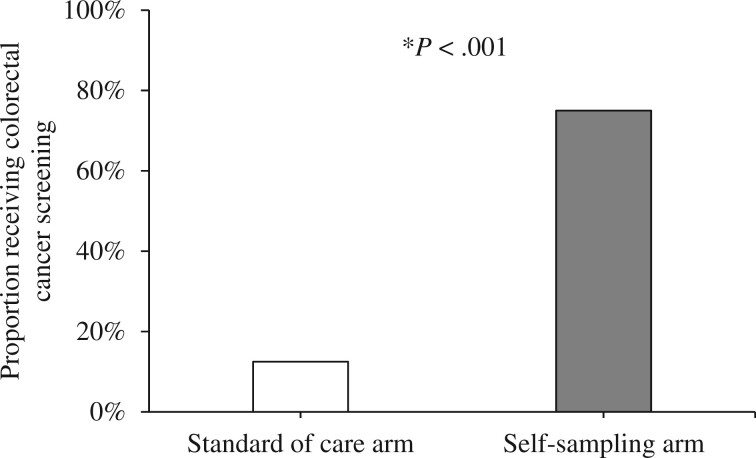
Proportion of participants who received colorectal cancer screening during the 10-week follow-up period, by study arm (n* *=* *48; 24 participants in each arm). Receipt of screening was measured at the follow-up survey; all participants with missing data were assumed to not have received screening. *P < .001 for comparison between standard of care and self-sampling arm.

Colorectal cancer screening was 13% (3 of 24) in the standard-of-care arm and 75% (18 of 24) in the self-sampling arm (Figure 2). Screening was higher in the self-sampling arm than the standard-of-care arm (OR = 31.32, 95% CI = 5.20 to 289.33).

Among unscreened participants, 1 reported scheduling health-care appointments to receive colorectal cancer screening (n* *=* *1 in standard-of-care arm), and 1 was in the process of scheduling this appointment (n* *=* *1 in standard-of-care arm). Few of the unscreened participants intended to get screened within the next year (standard-of-care arm: 22%, 4 of 18; self-sampling arm: 0%, 0 of 3).

#### Uptake of cervical cancer screening

**Figure 3.**
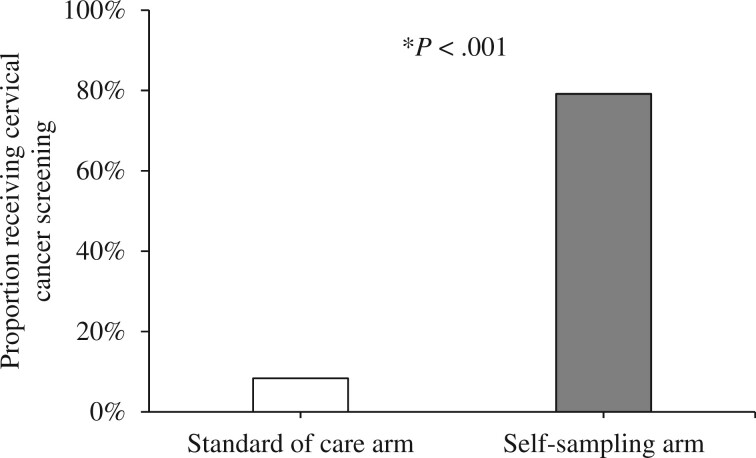
Proportion of participants who received cervical cancer screening during the 10-week follow-up period, by study arm (n* *=* *48; 24 participants in each arm). Receipt of screening was measured at the follow-up survey; all participants with missing data were assumed to not have received screening. *P < .001 for comparison between standard of care and self-sampling arm.

Cervical cancer screening was 8% (2 of 24) in the standard-of-care arm and 79% (19 of 24) in the self-sampling arm (Figure 3). Screening was higher in the self-sampling arm than the standard-of-care arm (OR = 72.03, 95% CI = 9.15 to 1141.41).

Among unscreened participants, 4 reported that they had scheduled health-care appointments to receive cervical cancer screening (n* *=* *3 in standard-of-care arm, n* *=* *1 in self-sampling arm), and 1 was in the process of scheduling this appointment (n* *=* *1 in standard-of-care arm). Despite these reports, few of the unscreened participants intended to get screened within the next year (standard-of care-arm: 16%, 3 of 19; self-sampling arm: 0%, 0 of 2).

### Self-sampling outcomes

Most (71%; 17 of 24) participants in the self-sampling arm returned the self-sampled fecal immunochemical test and HPV tools (Table 2). Among these participants, 24% (4 of 17) had abnormal results on colorectal cancer screening, and 18% (3 of 17) had abnormal results on cervical cancer screening.

**Table 2.** Cancer screening outcomes among participants in the self-sampling arm (n* *=* *24)

Cancer screening outcome	No. (%)
Colorectal cancer screening	
Returned self-sampling tool	
No	7 (29)
Yes	17 (71)
Abnormal findings on screening (n* *=* *17)	
No	13 (76)
Yes	4 (24)
Cervical cancer screening	
Returned self-sampling tool	
No	7 (29)
Yes	17 (71)
Abnormal findings on screening (n* *=* *17)	
No	14 (82)
Yes	3 (18)

### Psychosocial variables associated with screening

Across study arms, knowledge about cancer screening at baseline was positively associated with screening for colorectal cancer (OR = 2.38, 95% CI = 1.10 to 5.90 per 1-point increase in knowledge) and for cervical cancer screening (OR = 2.87, 95% CI = 1.29 to 7.60) during the study period (Table 3). No other relationships were detected between psychosocial variables and screening outcomes.

**Table 3.** Associations between baseline scores on psychosocial variables and cancer screening at follow-up^a^

Psychosocial variables	Any colorectal cancer screening OR (95% CI)	Any cervical cancer screening OR (95% CI)	Return of self-sampling tools OR (95% CI)
Health-care trust	0.76 (0.37 to 1.47)	0.73 (0.36 to 1.43)	0.37 (0.07 to 1.32)
Cancer fatalism	1.45 (0.45 to 4.67)	1.97 (0.59 to 6.51)	1.32 (0.18 to 9.38)
Cancer screening knowledge	2.38 (1.10 to 5.90)	2.87 (1.29 to 7.60)	7.79 (1.52 to 142.29)

a Outcomes of any colorectal cancer screening and any cervical cancer screening were assessed among all participants who completed the follow-up survey (n* *=* *42), using intent-to-treat analysis. Outcome of return of self-sampling tools was assessed among all participants randomly assigned to the self-sampling arm (n* *=* *24). CI = confidence interval; OR = odds ratio.

## Discussion

Overall, our findings demonstrate the feasibility, acceptability, and potential impact of self-sampling tools for increasing uptake of cervical and colorectal cancer screening. Participants were statistically significantly more likely to screen for both cancers if they received a package of low-literacy educational materials and self-sampling tools for fecal immunochemical and HPV testing compared with standard of care. Abnormal results on the self-sampled cancer screening tests were relatively common, which underscores the need for linkages to follow-up care after patients complete self-sampling tests.

The current study demonstrates the potential impact of self-sampling tools for improving cancer screening among patients in underserved communities. Uptake of screening was higher for participants who received educational materials and self-sampling tools than for participants who received standard of care (colorectal cancer: ≥63%; cervical cancer: ≥71%). This large difference in screening persisted even after accounting for whether unscreened participants had scheduled in-person health-care appointments to receive cancer screening. Underserved patients may view self-sampling tools as a method for re-engaging with primary care (36,37), which can facilitate access to other preventive services. These findings are highly relevant to ongoing national efforts to expand access to self-sampling tools for cancer screening, including the 2022 President’s Cancer Panel Report (15) and the National Cancer Institute’s Cervical Cancer “Last Mile” Initiative (38), which is focused on accruing the evidence to support regulatory approval of HPV self-sampling. The current study provides strong evidence for expanding access to self-sampling tools to increase screening and reduce geographic disparities in cancer outcomes.

The rate of returning self-sampling tools for cancer screening was higher (71%) in the current study compared with previous studies. Other community-based studies have achieved return rates of 19%-37% for colorectal cancer (39-41) or 26%-78% for cervical cancer (42-44). The high return rate in the current study could be related to unique characteristics of our sample of rural patients and federally qualified health centers; for example, the patient population generally did not include migrant farm workers, frontier or remote locations, or hospital-affiliated practices (39). Other explanations for the high return rate include the small sample size, the COVID-19 pandemic [which was associated with reductions in cancer screening uptake (45)], and self-selection of motivated patients into the trial (44).

In addition, the rate of abnormal results on cancer screenings in the current study was high. For colorectal cancer, 24% of participants in the current study had abnormal findings on their fecal immunochemical test compared with less than 10% in previous studies (46-48). For cervical cancer, 18% of participants had abnormal findings on their self-sampled HPV test, which is approximately equal to other studies, which generally find less than 20% (19,20,24,42). [It should be noted that other studies of the self-sampled HPV test generally recruit participants aged 30-65 years compared with 50-65 years in the current study, and HPV positivity tends to be lower among older patients (49); that is, we might expect a higher rate of abnormal results on self-sampled HPV tests among patients aged 30-65 years than what we observed in the current study.] Therefore, the rate of abnormal results in the current study suggest that this patient population would benefit from cancer screening outreach and navigation. For study participants who had abnormal findings on their self-sampled screenings, a physician at the federally qualified health center called them to discuss appropriate next steps; the physician was able to contact all of these participants and create a clinical plan for follow-up care.

We also found that cancer screening knowledge at baseline was associated with screening during the follow-up period. These findings provide further support for knowledge being an important facilitator of cancer screening (50,51). The intervention materials included low-literacy educational materials to improve cancer screening knowledge; however, knowledge measured at follow-up was not associated with screening (data not shown). Additional research is needed to parse the timing of patient education, cancer screening knowledge, and screening behaviors and to determine which educational messages can best motivate patients to complete self-sampling. Cancer screening education may be particularly important for HPV self-sampling, given the novelty of this test modality.

Multicancer screening approaches are often discussed among cancer control advocates, but there is a paucity of data on the potential impact and efficiency of combined screening interventions. Our results are among the first to evaluate the impact of combining screening for multiple cancers using self-sampling. The high level of participant engagement and increased screening with self-sampling are especially important for addressing barriers to care among rural populations. Although sample sizes were small in our feasibility study, we used a randomized design, validated measures, and evaluation of potential impact measures for future implementation of larger-scale intervention studies.

The response rate for our study was low (approximately 6%), which suggests that additional supports are needed to improve participation beyond the low-cost methods we used for recruitment. Currently, self-sampling is not a US Food and Drug Administration–approved modality for HPV testing, but our study provides useful data to support future implementation once approved in the United States. We used self-report to measure receipt of cancer screening during the follow-up period, which is subject to some bias (52); in future studies, we hope to verify self-reported screening with electronic health record data. Further, the follow-up period for measuring receipt of cancer screening was relatively short (ie, 10 weeks), which could have prevented some participants from receiving cancer screening, especially if they chose to receive screening in an in-person health-care setting. This limitation could have introduced bias in terms of uptake of screening among participants in the standard-of-care arm; however, it is notable that few of the unscreened participants reported scheduling an appointment for screening between study initiation and follow-up data collection.

Because many patients living in underserved communities experience barriers to health-care access, self-sampling may offer improved access to cancer screenings. Furthermore, the ease and privacy of self-sampling may improve acceptance of these screenings (53). Primary care clinicians should be prepared to discuss self-sampling for cancer screening with their patients, especially those who express reservations about other forms of cancer screenings or who have to travel long distances to access care.

Descriptively, we evaluated the external validity of our findings by comparing demographic characteristics of the study sample with characteristics of other select groups. First, comparing the sample to the population in the underlying counties, we found that 83% of participants were non-Hispanic White (compared with 90% of residents in the underlying counties), and 67% of participants earned less than $50 000 (compared with 45% in the underlying counties). Thus, participants were similar to residents of the underlying counties in terms of race and ethnicity but tended to have lower income. These patterns may have emerged because of study design choices (eg, partnering with federally qualified health centers, which serve low-income patients). Second, comparing the sample with the patients we assessed for eligibility, we found that enrolled participants were older than patients assessed but deemed ineligible (mean age = 55.8 vs 51.7 years, respectively; *P* < .001). Of the assessed patients, 31 were deemed ineligible for participation because they were aged younger than 50 years (which explains the age difference across groups); however, many of these patients would have been eligible for cervical cancer screening, illustrating a high level of interest in the self-sampling option for that test. A third comparison of interest would be of enrolled participants compared with invited patients, but because of patient privacy concerns, the federally qualified health center partner did not share data on that group; it would be informative to understand differences in patients who responded vs did not respond to study invitations to assess potential implications for scale-up of self-sampling modalities. Thus, the external validity of our findings may be limited by characteristics of the study sample (ie, lower income and older) coupled with the overall low response rate (approximately 6%), which could introduce bias in terms of willingness to use a self-sampling option for cancer screening. Future studies should continue to explore feasibility and potential effects of self-sampling with other settings, populations, and outreach strategies.

This study adds important findings to our evidence base about improving screening uptake and adherence among underserved communities. Although many studies have shown increased cervical cancer screening through self-sampling in global settings (20,54), this feasibility trial strongly suggests that self-sampling may promote screening for multiple cancers.

Providing a package of low-literacy educational materials and self-sampling tools for fecal immunochemical and HPV testing to underserved patients facilitated very high uptake of colorectal and cervical cancer screenings compared with standard-of-care procedures. These findings have implications for efforts to expand access to self-sampling tools. In the long term, these tools can increase cancer screening, decrease cancer burden, and decrease geographic disparities in cancer outcomes experienced by patients from underserved communities.

## Data Availability

Data are available upon request to the first author.
